# Impact of Supplemented Nutrition on Semen Quality, Epigenetic-Related Gene Expression, and Oxidative Status in Boars

**DOI:** 10.3390/ani14223297

**Published:** 2024-11-15

**Authors:** Jovan Blagojević, Zoran Stanimirović, Uroš Glavinić, Slobodanka Vakanjac, Željko Radukić, Milorad Mirilović, Milan Maletić

**Affiliations:** 1 Department of Biology, Faculty of Veterinary Medicine, University of Belgrade, 11000 Belgrade, Serbia; jovan.blagojevic@vet.bg.ac.rs (J.B.);; 2 Department of Reproduction, Fertility and Artificial Insemination, Faculty of Veterinary Medicine, University of Belgrade, 11000 Belgrade, Serbiamaletic@vet.bg.ac.rs (M.M.); 3 Animal Husbandry and Veterinary Centre “Velika Plana”, 11320 Velika Plana, Serbia; 4 Department of Economics and Statistics, Faculty of Veterinary Medicine, University of Belgrade, 11000 Belgrade, Serbia

**Keywords:** boar sperm, CASA, epigenetic-related genes, oxidative stress, nutrition, supplementation

## Abstract

This study investigates the impact of nutritional supplementation on semen quality, epigenetic-related gene expression, and oxidative status in boars. Thirty boars were divided into a control group and a treatment group receiving Espermaplus (a supplement containing various vitamins, amino acids, omega-3 fatty acids, and trace elements with antioxidant properties). The experiment was performed for 12 weeks. Semen samples were collected at four moments: before starting the supplementation and after 3 weeks, 8 weeks, and 12 weeks. Spermatozoa concentration, motility, and kinematics were assessed using the CASA system. The measured parameters included curvilinear velocity—VCL; straight-line velocity—VSL; average path velocity—VAP; curvilinear distance—DCL; straight line distance—DSL; distance of average path—DAP; amplitude of lateral head displacement—ALH; beat-cross frequency—BCF; and head activity—HAC. Moreover, superoxide dismutase (SOD) and glutathione peroxidase (GPx) activity in seminal plasma, as well as the concentration of thiobarbituric acid reactive substances (TBARS), were measured to assess oxidative stress levels in boar’s seminal plasma. The expression of epigenetic-related genes such as Protamine 1 (*Prm1*), Protamine 2 (*Prm2*), and DNA-methyltransferase 3 alpha (*Dnmt3a*) were evaluated using real-time PCR. The treated group showed a significant increase in spermatozoa concentration (*p* = 0.003), total motility (*p* = 0.001), and progressive motility (*p* = 0.002) after 12 weeks compared to the control group. Kinematic parameters such as VCL, VSL, and VAP were also significantly higher (*p* < 0.001; *p* = 0.028; *p* < 0.001, respectively) in the treated group by the end of the experiment. SOD and GPx activities were consistently higher (*p* < 0.01; *p* < 0.001, respectively) in the treated group, indicating enhanced antioxidative capacity. TBARS levels as an indicator of lipid peroxidation and oxidative damage were significantly lower (*p* < 0.01) in the treated group by the end of the study. Significant changes were observed in the expression of epigenetic-related genes. The supplementation of boar diets with Espermaplus significantly improved semen quality, reduced oxidative stress, and had an impact on the expression levels of certain epigenetic-related genes, suggesting that dietary antioxidants and bioactive compounds can enhance boar semen.

## Simple Summary

Improving boar semen quality is essential for successful artificial insemination due to its direct impact on fertility rate and litter size, making it very important for efficient reproduction in pig farming. Boar sperm is highly sensitive to oxidative stress, which damages sperm cells and decreases fertility. This study is focused on finding whether dietary supplementation can enhance the quality of boar semen, preserve sperm from oxidative damage, and affect the expression of epigenetic-related genes by examining the impact of particular nutrients with antioxidant effects. Key findings revealed that the supplemented group showed improved sperm concentration, motility, and kinematics, with enhanced antioxidative capacity, through increased enzyme activity in seminal plasma and lower lipid peroxidation markers. Changes in epigenetic-related gene expression levels also indicated positive impacts on genetic stability, which could enhance fertility. These results imply that dietary supplementation could improve reproductive performance in boars.

## 1. Introduction

Pig breeding is of great importance for the economy of many countries. To improve productivity and profitability in pig farming, artificial insemination (AI) has been increasingly used during the last few decades [1]. For AI, it is necessary to provide high-quality reproductive material from boars, which requires constant improvement procedures and technologies in semen processing. Boar semen quality is crucial for successful reproduction in pig farming, as it directly influences fertility rates and litter size. For AI in the pig industry, diluted and liquid-preserved semen is primarily used, stored at a temperature of 15–18 °C, no longer than 5–10 days [2]. Research has shown that after the third day of storage, the sperm quality decreases, as does its fertility [3]. Therefore, efforts are constantly being made to increase the quality of raw semen and to prolong its sustainability. One of the ways to achieve this is through diet supplements. Dietary supplementation can protect spermatozoa from oxidative damage and extend their lifespan (Pascoal et al., 2022) [4]. This is important because boar sperm is highly sensitive to oxidative damage (Lee et al., 2020) [5]. Several antioxidants used as dietary supplements have been shown to significantly improve the quality parameters of boar semen, such as vitamin C, zinc, selenium, and vitamin E [6,7,8].

Antioxidants are generally defined as compounds that reduce, scavenge, and suppress reactive oxygen species (ROS) production and plasma membrane lipid peroxidation. They are well known as beneficial for human and animal health, but research is still needed to find optimal formulations that can improve the reproductive characteristics of breeding stock [9,10]. ROS are normally produced in spermatogenesis [11] and their concentration plays an essential function in spermatogenesis, which is particularly important during spermiogenesis and in processes involving spermatozoa capacitation and acrosomal reaction [12]. The increased production of ROS can lead to cell and tissue damage, leading to the activation of antioxidant enzymes to preserve homeostasis [13]. The activity of antioxidant enzymes can serve as a useful marker of the level of oxidative stress, as well as the health status of boars [14]. Antioxidant enzymes accumulated in seminal plasma have a vital function in decreasing ROS-induced damage. The most potent antioxidant enzymes found in semen are superoxide dismutase (SOD) and glutathione peroxidase (GPx). SOD activity grows depending on the oxygen level in vivo, and its metabolic role is demonstrated by the conversion of superoxide radicals (O^2^) into hydrogen peroxide (H_2_O_2_;) [15]. GPx binds to the plasma membrane of spermatozoa and protects them from the detrimental impacts of peroxides [16]. Spermatozoa are exposed to oxidative stress at all stages of development within the male reproductive tract. Also, damage to spermatozoa occurs to a large extent after ejaculation, during the manipulation of semen [17].

The spermatozoon plays a critical role in delivering genetic material to the egg cell and initiating zygote formation, thus featuring a distinct chromatin composition compared to somatic cells [18,19]. It is the replacement of histones by protamines during spermiogenesis that leads to a denser packaging of DNA, so the interaction between protamines and DNA can be considered a form of epigenetic regulation specific to spermatozoa, but not to somatic cells [20]. Epigenetic modifications during spermatogenesis may contribute to impaired sperm function and fertilization efficiency. The adequate establishment of epigenetic patterns is believed to be key to maintaining proper sperm function, and research shows that oxidative stress significantly affects the degree of DNA methylation, as well as other epigenetic modifications [21]. However, there is a lack of data in the literature proving the effects of supplemented nutrition on epigenetic-related gene expression and oxidative stress in spermatozoa under in vivo conditions. Keeping in mind that nutrition strongly influences boar semen, this research aims to examine the impact of supplemented nutrition on boar semen quality and the expression of epigenetic-related genes together with monitoring oxidative stress parameters. To our knowledge, this is the first study of diet supplementation investigation on epigenetic-related gene expression in boar sperm.

## 2. Material and Methods

### 2.1. Animals and Experimental Design

Thirty boars of different breeds (large white, landrace, pietren), two years old, located in the Animal Husbandry and Veterinary Center “Velika Plana” in Velika Plana (Serbia) were included in the research. The experiment was conducted from October to December 2021. The animals were nourished with a standard commercial breeder mixture PN-16, (registration number: RS 30-074), complete mixture for breeding boars, Pantelic d.o.o., Kraljevo, Serbia) consisting of cereals, soybean meal, mineral mixture, and common salt to meet their nutritional requirements [22]. The meals were thoroughly mixed immediately before feeding, ensuring that each boar received 30 g of supplement per kg of the feed (75 g/boar/day). Boars were fed a total mixed ration twice daily, consuming 2.5 kg per day, with ad libitum access water.

The boars were randomly divided into two equal groups with 15 boars in each, one of which was the control group (CON), fed standard breeder mixture (PN-16) during the whole experiment. The other group consisted of boars that were fed the standard breeder mixture but with the addition of a supplement (Espermaplus, Magapor, Zaragoza, Spain)—the treated group (ESP). Espermaplus is a mixture of liposoluble (A, D, E, and K vitamins) and water-soluble vitamins (B1, B2, B6, B12, and C vitamins; biotin; folic acid; nicotinic acid; pantothenic acid; and choline), omega-3 fatty acids, amino acids (lysine, methionine, threonine, and tryptophan), and oligo-elements (zinc, selenium, copper, iron, copper, manganese, and iodine), many of which have antioxidant properties. The experiment lasted 12 weeks, keeping in mind that average spermatogenesis in boars lasts about 6 weeks [23,24]. The effect of supplementation on the parameters of semen quality, oxidative stress, and the expression of epigenetic-related genes was monitored at certain intervals from the beginning of supplementation. Semen sampling was performed four times during the investigation period: before starting the supplementation (P1 moment), after three weeks (P2 moment), after 8 weeks (P3 moment), and after 12 weeks (P4 moment). The first sampling was taken before supplementation (time P1), and the second was taken three weeks after the start of supplementation (time P2), or roughly halfway through the spermatogenesis cycle, to observe the impact of supplementation on spermatozoa during their maturation. The P3 moment was 8 weeks after the start of supplementation, so the sperm collected at this time was produced during supplementation. The P4 moment was 12 weeks after the start of the supplementation, covering two entire cycles of spermatogenesis. Semen collection was performed by deploying a manual penis fixation method [25]. The first gelatinous fraction was discarded and the second sperm-rich fraction was collected into a semen collector covered with sterile gauze. Immediately after collection, 2 mL of semen was taken and centrifuged (1000× *g* for 15 min) to obtain seminal plasma. Collected plasma samples were stored at −20 °C and used for oxidative stress analyses. Another aliquot of 1 mL of ejaculate was used for RNA extraction, while the rest of ejaculate was diluted with a Duragen extender (Magapor, Spain) and used for semen quality parameters evaluation.

### 2.2. Computer Assisted Sperm Analysis

Sperm concentration, motility, and kinematic parameters were determined using the Computer-Assisted Sperm Analysis (CASA) system (Minitube, AndroVision, Tiefenbach, Germany). Before the analysis, semen samples were previously extended with Duragen (Magapor, Spain) and heated to 38 °C for 10 min. After heating, 2.7 μL of the sample was added to the Leja chambers (GN Nieuw Vennep, The Netherlands). Ten microscopic fields with between 1500 and 2000 spermatozoa were analyzed on a phase-contrast microscope (Motic BA310, Barcelona, Spain, Page 4/21) with a built-in heating plate. AndroVision (Minitube, Tiefenbach, Germany) software version 1.1.4 was used to automatically analyze motility characteristics [26]. The CASA system measured the following semen quality parameters: sperm concentration, total and progressive sperm motility, and sperm kinematics parameters including curvilinear velocity—VCL [µm/s]; straight-line velocity—VSL [µm/s]; average path velocity—VAP [µm/s]; curvilinear distance—DCL [µm]; straight line distance—DSL [µm]; distance of average path—DAP [µm]; amplitude of lateral head displacement—ALH [µm]; beat-cross frequency—BCF [Hz]; and head activity—HAC [rad]. CASA software (version 1.1.4) settings recommended by Minitube were as follows: field of view of 565 × 565 µm with a pixel size of 0.55 µm; object features area 5–80 µm; automatic threshold with Offset 20; a cutoff value for immotile cells was ALH < 1 µm and VCL < 24 µm/s or HAC < 0.033 rad; spermatozoa with VSL < 24 µm/s and VCL > 48 µm/s were considered locally motile (otherwise considered progressively motile). Those with a radius > 10 and <30 and rotation > 7% were considered to have circular motility. Spermatozoa with a VCL < 120 µm/s were considered slow motile; otherwise, if VCL > 120 µm/s, they were considered fast motile.

### 2.3. Oxidative Stress Parameters Assesment

The activity of antioxidant protection enzyme superoxide dismutase (SOD) and glutathione peroxidase (GPx) was determined, as well as the concentration of thiobarbituric acid reactive substances (TBARS) in seminal plasma.

SOD activity was measured indirectly by monitoring the degree of inhibition of adrenaline autoxidation to adrenochrome in the alkaline medium according to Misra and Fridovich [27] and Yelumalai et al. [28]. The reaction mixture containing seminal plasma, 0.05 mol/L carbonate buffer (pH = 10.2), and 0.l mmol/L EDTA was incubated at 25 °C for three minutes after the addition of 20 mmol/L adrenaline, and the rate of adrenochrome formation was monitored at a wavelength of 480 nm for 3 min in a spectrophotometer (UV–Vis Spectrophotometer BK-S390 BIOBASE, Shandong, China). The activity of this enzyme, expressed in international units (U), was obtained by measuring the absorbance of the adrenaline oxidation product in the presence of the enzyme. The amount of enzyme required to reduce the rate of autoxidation by 50% in an alkaline environment was established as the unit of enzyme activity. The result of SOD activity is expressed as units per milliliter of plasma (U/mL).

The GPx activity was determined using a method based on the reduction of H_2_O_2_ in the presence of reduced glutathione, which is transformed into glutathione disulfide under the influence of GPx [29,30]. The reaction mixture consisting of 0.1 M phosphate buffer (pH 7.4), 2 mM GSH, 10 mM sodium azide, 1 mM H_2_O_2_, and seminal plasma was terminated by adding 5% trichloroacetic acid, and the colored complex with Ellman’s reagent (5.5-dithiobis-2-nitrobenzoic acid, DTNB) was read at 412 nm. GPx activity was expressed in U/mL of seminal plasma.

To assess lipid peroxidation, the level of TBARS was measured using the thiobarbituric acid (TBA) test. Thiobarbituric acid reactive substances, as a specific product of lipid peroxidation, react with TBA. The method is based on measuring the purple color, which is produced as a product of the reaction of the TBARS and TBA complex, with a spectrophotometer (UV–Vis Spectrophotometer BK-S390 BIOBASE) at a wavelength of 535 nm [31,32]. The concentration of TBARS was expressed in units per milliliter of plasma (U/mL).

### 2.4. Epigenetic-Related Genes Expression

The expression of the genes for Protamine 1 (*Prm1*), Protamine 2 (*Prm2*), DNA-methyltransferase 3 alpha (*Dnmt3a*), DNA-methyltransferase 3 beta (*Dnmt3b*), JmjC domain-containing histone (*Jhdm2a*), K (lysine) acetyltransferase 8 (*Kat8*), and insulin-like growth factor 2 (*IGF2*) were assessed according to the protocol of Zeng et al. [33]. Keeping in mind that the Glyceraldehyde-3-phosphate dehydrogenase (*GAPDH*) gene showed the most stable expression in the boar spermatozoa, it was used as an endogenous control [34]. To exclude possible contamination by leukocytes, epithelial cells, and testicular germ cells, *CD45*, *c-kit*, and *E-cadherin* genes were used as an indicator of the presence of this cell [33]. The expression of epigenetically related genes was monitored in the P3 and P4 moments in both CON and ESP groups.

#### 2.4.1. RNA Extraction and cDNA Synthesis

After the centrifugation of 1 mL of semen (3400× *g* at 4 °C for 5 min), the pellet was resuspended with somatic cell lysis solution (SCLS) containing 0.1% sodium dodecyl sulfate and 0.5% Triton X in distillate H_2_O [35,36]. This suspension was incubated on ice for 15 min in order to lyse the somatic cells [34]. Therefore, the absence of expression of somatic and germ cell-specific genes (*CD45, c-kit,* and *E-cadherin*) indicates that the lysis of these cells was successfully performed at this step. Further on, total RNA was extracted using a commercial TRIzol LS Reagent kit (Invitrogen, Carlsbad, CA, USA) according to manufacturer’s instructions. The quality and quantity of extracted RNA were checked using a BioSpec-nano spectrophotometer (Shimadzu Scientific Instruments, Kyoto, Japan). RNA was then converted into cDNA using a commercial FastGene Scriptase II cDNA Kit (Nippon Genetics, Düren, Germany) according to the manufacturer’s protocol.

#### 2.4.2. Real-Time PCR (q-PCR)

Real-time PCR was performed on a Rotor-Gene Q 5plex machine (Qiagen, Valencia, CA, USA). The amplification was performed in a 20 µL reaction mixture with “FastGene^®^ IC Green 2 × qPCR Universal Mix” (Nippon Genetics, Düren, Germany) according to the manufacturer’s instructions and with selected set of primers (Table 1). Gene expression levels were determined using 2^−ΔCt^ method. Briefly, the average quantity of each gene of interest was normalized according to the Ct value of the endogenous control—*GAPDH* gene (ΔCt = Ct _gene_ − Ct*_GAPDH_*).

### 2.5. Data Analysis

All obtained data were processed in GraphPad Prism 10.0 (GraphPad Software Inc., San Diego, CA, USA). In the first step, the normality of the obtained data was examined using the Shapiro–Wilk test. The results for sperm motility parameters and indicators of oxidative stress have a normal distribution (*p* > 0.05), while the data related to gene expression in spermatozoa showed a deviation from the normal distribution (*p* < 0.001). A two-way analysis of variance was performed to determine the effect of treatment (control and supplemented with Espermaplus), period (P1, P2, P3, and P4 moments), and treatment × period interaction on the parameters of sperm motility and indicators of oxidative stress in boars. Subsequently, a post hoc test (Tukey) was used to determine significant differences between pairs of average characters evaluated between treatments. On the other hand, the significance of differences in gene expression between the groups studied was examined using the non-parametric Mann–Whitney U test. Significance was declared at *p* < 0.05, *p* < 0.01, and *p* < 0.001, and a tendency was acknowledged at 0.05 ≤ *p* < 0.10.

## 3. Results

### 3.1. Semen Quality Parameters

The boars’ semen quality was monitored through the parameters of sperm motility, kinematics, and concentration (Supplementary Table S1). There were no significant differences (*p* > 0.05) between the experimental groups at the beginning of the study (before starting the supplementation—P1) regarding all the monitored parameters of semen quality, which indicates the uniformity of the experimental groups at the beginning of the experiment.

Sperm concentration in the ESP group (supplemented with Espermaplus) was significantly higher in P3 (*p* = 0.041) and P4 (*p* = 0.003) compared to the P1 moment, while there were no significant differences (*p* > 0.05) among the moments within the control (CON) group. When comparing experimental groups with each other, in the P4 moment, sperm concentration was significantly higher (*p* = 0.044) in the ESP group compared to the CON group, while in the previous three moments (P1, P2, and P3) there were no significant differences (*p* > 0.05). On the other hand, total motility (TM) and progressive motility (PM) were significantly affected by the treatment (*p* = 0.007 for TM; *p* = 0.030 for PM) and experimental period (*p* < 0.001). Thus, TM was significantly higher in the ESP group compared to the CON group in both P3 (*p* = 0.019) and P4 (*p* = 0.001) moments, while such differences between these groups in the PM were detected only in the P4 moment (*p* = 0.002). In addition, both TM and PM in the ESP group were significantly lower in the P1 moment compared to the P2 moment (*p* = 0.044 for TM; *p* = 0.046 for PM), the P3 moment (*p* = 0.006 for TM; *p* = 0.004 for PM), and the P4 moment (*p* < 0.001 for both TM and PM), indicating an increasing trend for these parameters in a supplemented group of boars.

Sperm kinematic parameters showed a tendency to increase under the influence of treatment. Straight-line velocity (VCL) was affected by the experimental period (*p* < 0.001). The VCL in the ESP group was significantly higher in the P4 moment compared to previous moments (P1, *p* < 0.001; P2, *p* < 0.001; P3, *p* = 0.035); moreover, the VCL of samples from the ESP group in the P4 moment was higher (*p* = 0.021) compared to the CON group at that moment (P4). Contrarily, no significant differences were detected (*p* > 0.05) in VCL among all moments in the CON group. Straight-line velocity (VSL) was affected by treatment (*p* = 0.040) and the examined period (*p* < 0.001). The VSL parameter in the ESP group was higher in the P4 moment compared to the P1 moment (*p* = 0.028), while in the CON group, the value of this parameter was lower in P1 compared to P2, P3, and P4 (*p* = 0.019; *p* = 0.003; *p* < 0.001; respectively). Average path velocity (VAP) was affected by treatment (*p* = 0.047) and period (*p* < 0.001). The VAP values in the ESP group were significantly higher in the P4 moment compared to the other moments (P1, *p* < 0.001; P2, *p* = 0.002; P3, *p* = 0.026), while in the CON group, the values of this parameter were significantly higher only in the P4 moment compared to the P1 moment (*p* = 0.045).

The curvilinear distance (DCL) was significantly (*p* < 0.001) affected by the period. In the ESP group, the DCL value was significantly higher in the P3 moment compared to the P2 moment (*p* = 0.012), in the P4 moment compared to the P1 moment (*p* < 0.001), and the P2 moment (*p* < 0.001). In the CON group, higher DCL values were observed in the P4 moment compared to the P1 moment (*p* = 0.007) and P2 moment (*p* = 0.010). Straight line distance (DSL) was affected by the examined period (*p* = 0.003). The DSL parameter was lowest in the P1 moment, being lower than in the P2, P3, and P4 moments (*p* < 0.001, *p* = 0.039, *p* = 0.004, respectively). Moreover, it was higher in the P2 moment compared to the P3 moment (*p* = 0.018) and P4 moment (*p* = 0.032). In the CON group, values for this parameter were significantly higher only in the P4 moment compared to the P1 moment (*p* = 0.029). Distance of average path (DAP) was affected by period (*p* < 0.001). In the ESP group, the DAP value was significantly higher in the P4 moment compared to the P1, P2, and P3 moments (*p* = 0.002, *p* < 0.001, *p* = 0.027, respectively), while in the CON group, the value of this parameter was significantly lower only in the P1 moment compared to the P4 moment (*p* = 0.002). According to statistical analyses, beat-cross frequency (BCF) was affected by the experimental period (*p* < 0.001). The BCF parameter in the ESP group had significantly higher values in the P4 moment compared to the P1 and P2 moments (*p* < 0.001, *p* = 0.011, respectively), while the value in the P3 moment was significantly higher compared to the P1 moment (*p* = 0.018). In the CON group, the BCF value was significantly lower in the P1 moment compared to the P2, P3, and P4 moments (*p* = 0.001, *p* = 0.018, *p* = 0.011, respectively). Thus, in the P4 moment, a significantly higher value was recorded in the ESP group compared to the CON group (*p* = 0.026). The amplitude of lateral head displacement (ALH) was affected by period (*p* < 0.001). In the ESP group, the value was significantly higher in the P4 moment compared to the P1 and P2 moments (*p* < 0.001) and in the P2 moment compared to P3 (*p* = 0.013). Also, in the CON group, the ALH value was significantly higher in the P4 moment compared to the P1 (*p* = 0.035) and P2 moments (*p* = 0.002). The head activity (HAC) was affected by period (*p* < 0.001). In the ESP group, a significantly higher value for the HAC parameter was observed in the P4 moment compared to the other examined moments (P1, *p* < 0.001; P2, *p* < 0.001; P3, *p* = 0.023). On the other hand, in the CON group, HAC values were significantly lower only in the P1 moment compared to the P4 moment (*p* = 0.004).

### 3.2. Oxidative Stress Parameters

**Figure 1:**
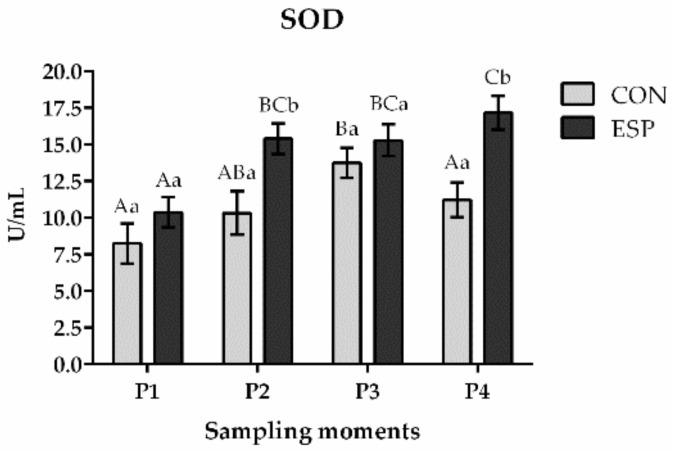
Effects of Espermaplus supplementation on superoxide dismutase (SOD) activity in sperm plasma. CON—control group; ESP—group supplemented with Espermaplus. P1—the day when Espermaplus supplementation started; P2—the day after three-week period of Espermaplus supplementation; P3—the day after the eight-week period of Espermaplus supplementation; P4—the day after the twelve-week period of Espermaplus supplementation. Different lowercase letters indicate statistically significant differences within the same group at different moments; Different uppercase letters indicate statistically significant differences between groups at the same sampling moment.

The ESP group had significantly higher SOD activity (Figure 1) compared to the CON group in the P2 and P4 moments (*p* < 0.01) while there were no significant differences in the P1 and P3 moments (*p* > 0.05). Our results revealed an increasing trend of SOD activity in the ESP group being higher in each sampling moment compared to the previous one (*p* < 0.05), while in the CON group, SOD activity was significantly lower in the P4 moment compared to the P3 moment (*p* < 0.05).

**Figure 2:**
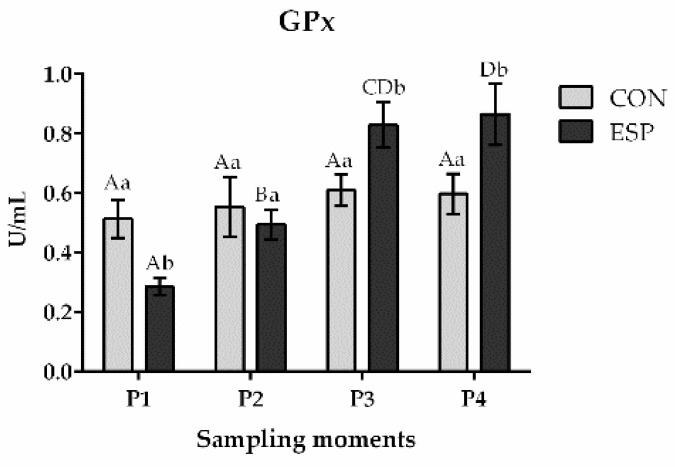
Effects of *Espermaplus* supplementation on glutathione peroxidase (GPx) activity in sperm plasma. CON—control group; ESP—group supplemented with *Espermaplus*. P1—the day when Espermaplus supplementation started; P2—the day after three-week period of Espermaplus supplementation; P3—the day after the eight-week period of Espermaplus supplementation; P4—the day after the twelve-week period of Espermaplus supplementation. Different lowercase letters indicate statistically significant differences within the same group at different moments; Different uppercase letters indicate statistically significant differences between groups at the same sampling moment.

The results showed a significant effect of the period (*p* < 0.01) and treatment x period interaction (*p* < 0.001) on the GPx activity (Figure 2). In addition, the activity of GPx was significantly higher in the ESP group compared to the CON group in both the P3 moment (*p* < 0.01) and P4 moment (*p* < 0.01). Interestingly, there is a noticeable trend of growth of GPx activity from the beginning (P1) to the end of the experiment (P4) in the ESP group of boars. GPx activity was significantly higher (*p* < 0.01) in the P2 moment than in the P1 moment within the ESP group. In addition, the activity of GPx was higher in the P3 moment compared to both P2 (*p* < 0.05) and P1 moments (*p* < 0.001) within the ESP group. Finally, the activity of GPx was also significantly higher in the P4 moment compared to both P2 (*p* < 0.05) and P1 (*p* < 0.001) moments. On the other hand, there were no differences (*p* > 0.05) in GPx activity within the CON group of boars during the experiment.

**Figure 3:**
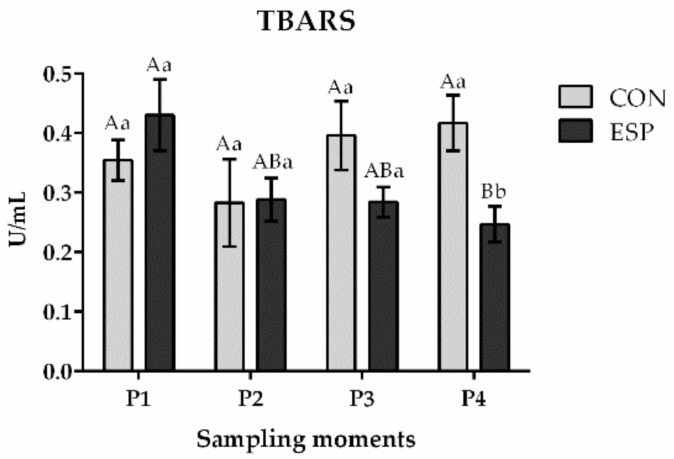
Effects of Espermaplus supplementation on thiobarbituric reactive substance (TBARS) concentration in sperm plasma. CON—control group; ESP—group supplemented with Espermaplus. P1—the day when Espermaplus supplementation started; P2—the day after three-week period of Espermaplus supplementation; P3—the day after the eight-week period of Espermaplus supplementation; P4—the day after the twelve-week period of Espermaplus supplementation. Different lowercase letters indicate statistically significant differences within the same group at different moments; Different uppercase letters indicate statistically significant differences between groups at the same sampling moment.

**Figure 4:**
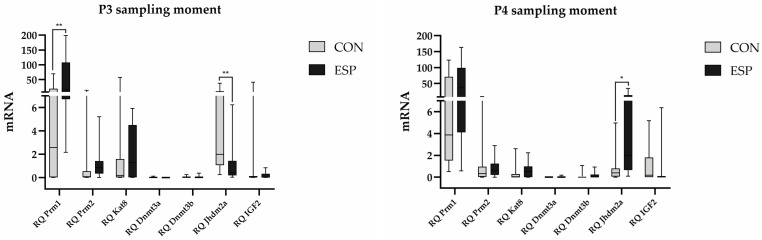
Epigenetic-related gene expression levels at the P3 moment (the day after the eight-week period of Espermaplus supplementation) and P4 moment (the day after the twelve-week period of Espermaplus supplementation) in the control (CON) and Espermaplus-supplemented (ESP) groups of boars. *—*p* < 0.05, **—*p* < 0.01.

The TBARS levels were significantly affected (Figure 3) by treatment × period interaction (*p* = 0.009) and tended to be affected by period (*p* = 0.078). Although no significant effect of treatment (*p* = 0.307) on TBARS level was found, a significantly lower level of TBARS was detected in the ESP group compared to the CON group in both P3 (*p* < 0.05) and P4 (*p* < 0.01) moments (Figure 4). Moreover, by observing the TBARS levels in the ESP group during the supplementation period, a trend of decreasing TBARS levels from the P1 moment to the P4 moment was obvious. Thus, the level of TBARS at the end of the supplementation period (P4 moment) was significantly lower (*p* = 0.02) than before the inclusion of the supplement in the boar diet (P1 moment). This was not observed in the CON group of boars where TBARS levels were more inconsistent and numerically higher at the end (P4 moment) than at the beginning of the experiment (P1 moment).

### 3.3. Epigenetic-Related Gene Expression Levels

**Figure 5:**
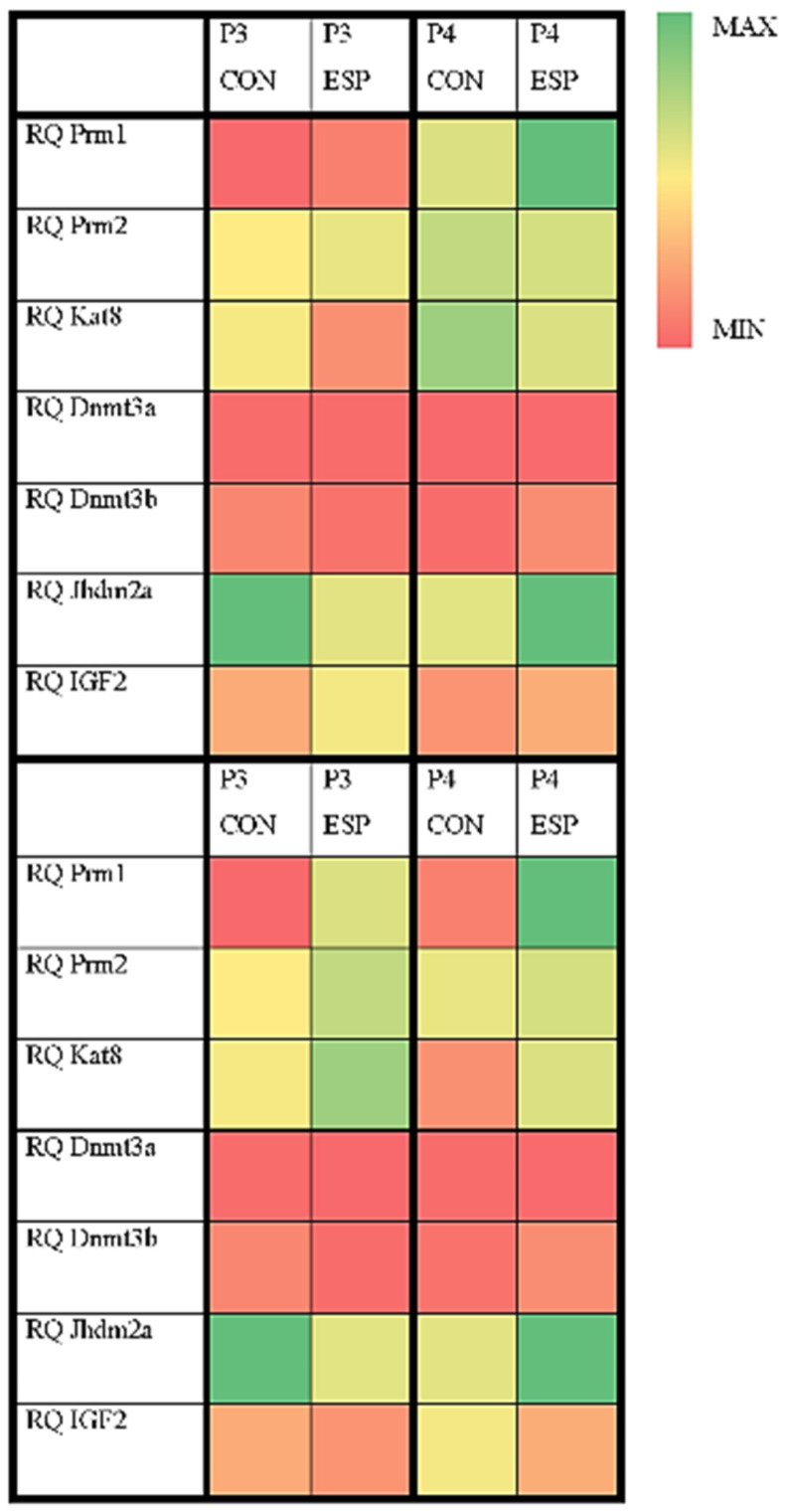
Heath map of median values for the relative gene expression levels (*Prm1*, *Prm2*, *Kat8*, *Dnmt3a*, *Dnmt3b*, *Jhdm2a*, *IGF2*) at different moments (P3 and P4) in the ESP and CON groups. CON—control group; ESP—Espermaplus-supplemented group; P3—the day after the eight-week period of Espermaplus supplementation; P4—the day after the twelve-week period of Espermaplus supplementation.

The results of expression levels of the epigenetic-related genes (Figure 4 and Figure 5) showed a significantly higher (*p* = 0.0043) expression of *Prm1*, as well as a significantly lower (*p* = 0.0086) expression of the *Jhdm2a* gene in the ESP group compared to the CON group in the P3 moment. Also, there was a noticeable trend of higher expression of *Prm2* (*p* = 0.0892) and *Dnmt3b* (*p* = 0.0814) genes in the ESP group in the same moment, even though they were not statistically significant. On the other hand, in the P4 moment, *Jhdm2a* gene expression levels were significantly higher (*p* = 0.0101) in the ESP group compared to the CON group. Expression of CD45, c-kit, and E-cadherin genes was not detected, indicating that the total RNA of boar spermatozoa was not contaminated with either somatic or germ cells. However, for the other monitored genes (*Prm2*, *Kat8*, *Dnmt3a*, *Dnmt3b*, and *IGF2* at the P3 moment; *Prm1*, *Prm2*, *Kat8*, *Dnmt3a*, *Dnmt3b*, and *IGF2* at the P4 moment), no statistically significant differences were recorded between the experimental groups.

## 4. Discussion

Dietary supplementation is a common practice in intensive livestock production when the demands for growth and reproduction are high because of high demands related to animal growth and reproduction [39]. Poor-quality nutrition results in reduced fertility and negative effects on production economics [40]. Therefore, supplementation through feed is meaningful if capable of improving semen quality by providing specific nutrients important for spermatogenesis. This kind of nutrition correction has an impact on the general health of the organism and the health of the acropodium, which is reflected in the reproductive performance of boars. Numerous studies have examined the effect of supplement-enriched diets on boar semen quality parameters [7,8,41]. It has been shown that antioxidants, omega-three fatty acids, and other bioactive compounds can affect sperm motility and viability, as well as DNA integrity [6,42]. In our research, the cumulative effect of the tested supplement showed significant beneficial effects by improving not only sperm concentration and total and progressive motility but also velocity parameters, especially VCL, VAP, DAP, and DCL. In this study, the sampling over 12 weeks was conducted to cover two cycles of spermatogenesis, considering that the average spermatogenesis lasts about 6 weeks [23,24]. Interestingly, the effect on the increase of kinematic parameters was mostly manifested only in the P4 moment (after 12 weeks), indicating the need for longer treatment/monitoring (>12 weeks) to achieve all the positive effects.

Food supplementation with vitamin E and selenium contributes to the preservation of spermatozoa morphology but also improves their motility and viability [43]. Antioxidant effects are believed to underlie the protective action of vitamin E and selenium against sperm damage and their effect on improving overall semen quality. Also, the addition of zinc in boar food increases sperm concentration, motility, and viability [7]. The activity of zinc in improving spermatogenesis is based on its effects on Leydig cells and sperm maturation [44]. Supplementation with omega-3 fatty acids led to a decrease in sperm velocity [45], while certain ingredients, such as linoleic acid, did not affect the quality parameters of boar semen [46]. In our study, the tested mixture improved semen quality, but since it consisted of a few supplements with well-known beneficial effects, further investigation of individual compounds is necessary to reveal their individual impact.

In our study, a noticeable increase in antioxidative capacity was recorded in the seminal plasma of boars that received the supplement. The influence of treatment is evident through the consistent increase of SOD and GPx activities. SOD is one of the essential antioxidant enzymes that are present in boar seminal plasma [47]. Although no direct effects of SOD levels on boar sperm fertility have been proven in vivo [47], maintenance of antioxidant protection enzyme activity is crucial in suppressing free radicals, which in high concentrations have detrimental effects on sperm cells [48]. Additionally, in the absence of catalase in semen, GPx is essential for protecting developing spermatozoa from the damaging effects of hydrogen peroxide [49]. Further on, TBARS, as an oxidative stress marker, showed reduced concentration in the treated group, contrary to the control group. The impact of oxidative stress on male reproductive function is mostly mediated by lipid peroxidation. Due to the susceptibility of boar spermatozoa to oxidative damage and the low level of antioxidant protection of seminal plasma, there is a need to increase the antioxidant capacity of boar semen [6]. During the maturation of spermatozoa, the majority of the cytoplasm is reduced, and the transcriptional and translational potential is lost, so the intracellular level of antioxidant enzymes is very low [48]. In this regard, seminal plasma is the main carrier of antioxidant protection of spermatozoa [50]. Walke et al. [51] also highlighted the role of oxidative stress in sperm cell damage and how antioxidant supplementation could mitigate this effect, similar to our findings.

Environmental stimuli, such as nutrition, are known to alter epigenetic regulation [52]. In our study, higher expression levels of *Prm1* and lower levels of *Jhdm2a* were observed in the supplemented group in the P3 moment (after 8 weeks). In addition, trends of increased expression of *Prm2* and *Dnmt3b* were also observed, although not statistically significant. After 12 weeks (P4 moment), the expression levels of *Jhdm2a* gene were higher in the supplemented group compared to the control group. *Jhdm2a* (JmjC domain-containing histone demethylation protein 2A) is a histone demethylase with an important role in transcriptional activation during spermatogenesis. Okada et al. [53] found that the Jhdm2a enzyme positively regulates the *Prm1* gene in round spermatids [53]. This enzyme plays a key role in the regulation of chromatin structure and gene expression through histone modification and is highly expressed in various spermatogenic stages through the spermatogenesis [53]. Having this in mind, we can conclude that in our study, longer supplementation induced upregulation of *Jhdm2a* gene expression (detected in the P4 moment) compared to samples taken a few weeks before (P3 moment). The detected upregulation in *Jhdm2a* gene in the P4 moment, which will lead to an increase of Jhdm2a enzyme level, contributes to better chromatin condensation and improvement of sperm quality. The changes in the expression of epigenetic-related genes observed in the supplemented (ESP) group in the P3 and P4 moments suggest that the tested supplement affects the expression of genes involved in spermatozoa development and function, which is in line with the previous findings of Pascoal et al. [4] and could be crucial for the oxidative status of semen as well as semen quality parameters. Previous studies have indicated that epigenetic markers are closely regulated during the process of spermatogenesis, so any alteration related to epigenetic regulation can lead to subfertility [54]. Further examination of the molecular mechanisms underlying epigenetic changes could contribute to a deeper understanding of how nutritional supplementation affects sperm biology. Moreover, further studies are needed during a more extended experimental period, including some additional time points to find out if the response changes over time.

## 5. Conclusions

In summary, the addition of the tested diet supplement improved boar semen quality as evidenced by increased sperm concentration, total and progressive motility, and sperm kinematic parameters; reduced oxidative stress detected through decreased levels of TBARS; and increased activity of SOD and GPx. The listed effects, together with changes in expression levels of some epigenetic-related genes, present a good basis for further research in order to reveal the specific mechanisms of supplements’ effects on boar semen.

## Figures and Tables

**Table 1:** List of used primers.

Primer	Sequence 5′–3′	Reference
*Prm1*	F: AGGAGGCGATGTTGTCCGAG R: ATTTCAGGCAGGAGTGCGGT	[37]
*Prm2*	F: AGTCCGAGTGAAAGTCCGCAG R: TGTGGCTCCTGTGTCTGTAGTGG	[34]
*Dnmt3a*	F: GCTTGTGTGTAAGGGACGTGA R: GGAATTTCCGCCTGCGTTTTG	[34]
*Dnmt3b*	F: AGGTCTCCAGCCTCCTAAGTT R: GTGTCTGAGCCATCTCCATCC	[34]
*Jhdm2a*	F: GCTCACTGCTGTCGGGTCT R: AAGGTGACGTTGGCGATGC	[38]
*Kat8*	F: CCATCCTCCACTTTGTCCCC R: CCAATGGTTGCAGCTTTCCC	[34]
*IGF2*	F: GTGGCATCGTGGAAGAGTG R: CCAGGTGTCATAGCGGAAGAA	[34]
*GAPDH*	F: ACTCACTCTTCTACCTTTGATGCT R: TGTTGCTGTAGCCAAATTCA	[33]
CD45	F: AGAATACTGGCCGTCGATGG R: GCTGAACGCATTCACTCTCCT	[33]
c-Kit	F: GTTGATGACCTCGTGGAATGC R: CTGCTACTGCTGTCATTCCTAAGG	[33]
E-cadherin	F: GAAGCACAGAATCCCCAAGTG R: GGCGTGTTTGTCTTCCATTTC	[33]

## Data Availability

Data are contained within the article.

## Supplementary Materials

The following supporting information can be downloaded at: https://www.mdpi.com/article/10.3390/ani14223297/s1, Table S1: Mean ± SE and ANOVA *p*-values for the effect of supplementation on boar sperm quality parameters.

## References

[1] Waberski D., Riesenbeck A., Schulze M., Weitze K.F., Johnson L. (2019). Application of preserved boar semen for artificial insemination: Past, present and future challenges. Theriogenology.

[2] Mellagi A.P., Will K.J., Quirino M., Bustamante-Filho I.C., Ulguim R.D.R., Bortolozzo F.P. (2023). Update on artificial insemination: Semen, techniques, and sow fertility. Mol. Reprod. Dev..

[3] Namuncura C., Sánchez R., Pezo F., Uribe P., Navarro P., Zambrano F. (2020). Rest days and storage of boar semen at 17 °C: Effect on motility and sperm concentration. Andrologia.

[4] Pascoal G.D.F.L., Geraldi M.V., Maróstica M.R., Ong T.P. (2022). Effect of paternal diet on spermatogenesis and offspring health: Focus on epigenetics and interventions with food bioactive compounds. Nutrients.

[5] Lee S.H., Kim Y.J., Kang B.H., Yun Y.S., Park C.K. (2020). The relationship between acrosome reaction and polyunsaturated fatty acid composition in boar sperm. Reprod. Domest. Anim..

[6] Galić I., Dragin S., Stančić I., Maletić M., Apić J., Kladar N., Spasojević J., Grba J., Kovačević Z. (2022). Effect of an Antioxidant Supplement Combination on Boar Sperm. Animals.

[7] Kaewma S., Suphappornchai S., Suwimonteerabutr J., Am-In N., Techakumphu M. (2021). Zinc supplementation improves semen quality in boars. Thai J. Vet. Med..

[8] Lugar D.W., Harlow K.E., Hundley J., Goncalves M., Bergstrom J., Stewart K.R. (2019). Effects of increased levels of supplemental vitamins during the summer in a commercial artificial insemination boar stud. Animal.

[9] Bansal A.K., Bilaspuri G.S. (2018). Effect of ferrous ascorbate on in vitro capacitation of crossbred cattle bull spermatozoa. Online J. Vet. Res..

[10] Betarelli R.P., Rocco M., Yeste M., Fernández-Novell J.M., Placci A., Azevedo Pereira B., Castillo-Martín M., Estrada E., Peña A., Zangeronimo M.G. (2018). The achievement of boar sperm in vitro capacitation is related to an increase of disrupted disulphide bonds and intracellular reactive oxygen species levels. Andrology.

[11] Das A., Roychoudhury S., Kesari K.K., Roychoudhury S. (2022). Reactive oxygen species in the reproductive system: Sources and physiological roles. Oxidative Stress and Toxicity in Reproductive Biology and Medicine.

[12] Dutta S., Henkel R., Sengupta P., Agarwal A., Parekattil S., Esteves S., Agarwal A. (2020). Physiological role of ROS in sperm function. Male Infertility: Contemporary clinical Approaches, Andrology, ART and Antioxidants.

[13] Huchzermeyer B., Menghani E., Khardia P., Shilu A. (2022). Metabolic pathway of natural antioxidants, antioxidant enzymes and ROS providence. Antioxidants.

[14] Pezo F., Yeste M., Zambrano F., Uribe P., Risopatrón J., Sánchez R. (2021). Antioxidants and their effect on the oxidative/nitrosative stress of frozen-thawed boar sperm. Cryobiology.

[15] Islam M.N., Rauf A., Fahad F.I., Emran T.B., Mitra S., Olatunde A., Shariati M.A., Rebezov M., Rengasamy K.R., Mubarak M.S. (2022). Superoxide dismutase: An updated review on its health benefits and industrial applications. Crit. Rev. Food Sci. Nutr..

[16] Fang Y., Zhong R. (2020). Effects of oxidative stress on spermatozoa and male infertility. Free Radical Medicine and Biology.

[17] Schulze M., Schröter F., Jung M., Jakop U. (2020). Evaluation of a panel of spermatological methods for assessing reprotoxic compounds in multilayer semen plastic bags. Sci. Rep..

[18] Rathke C., Baarends W.M., Awe S., Renkawitz-Pohl R. (2014). Chromatin dynamics during spermiogenesis. Biochim. Biophys. Acta, Gene Regul. Mech..

[19] Teves M.E., Roldan E.R. (2022). Sperm bauplan and function and underlying processes of sperm formation and selection. Physiol. Rev..

[20] Patankar A., Parte P., Singh R., Singh K. (2017). Sperm Chromatin Compaction and Male Infertility. Male Infertility: Understanding, Causes and Treatment.

[21] Menezo Y.J., Silvestris E., Dale B., Elder K. (2016). Oxidative stress and alterations in DNA methylation: Two sides of the same coin in reproduction. Reprod. Biomed. Online.

[22] National Research Council (2012). Nutrient Requirements of Swine.

[23] Almeida F.F., Leal M.C., França L.R. (2006). Testis morphometry, duration of spermatogenesis, and spermatogenic efficiency in the wild boar (*Sus scrofa scrofa*). Biol. Reprod..

[24] Kuhlgatz D.A., Kuhlgatz C., Aepli M., Schumann B., Grossfeld R., Bortfeldt R., Jakop U., Jung M., Schulze M. (2019). Development of predictive models for boar semen quality. Theriogenology.

[25] Paschoal A.F.L., Mellagi A.P.G., Ferrari C.V., Takeuti K.L., Oliveira G.D.S., Bernardi M.L., Ulguim R.D.R., Bortolozzo F.P. (2021). Adjusted method of penis fixation during boar semi-automatic semen collection aiming to reduce bacterial contamination. Reprod. Domest. Anim..

[26] Nedić S., Đurić M., Vakanjac S., Arsić S., Nedić S., Samardžija M., Borozan S. (2023). Relationship between biochemical parameters and paraoxonase 1 activity of boar seminal plasma and semen quality. Vet. Res. Commun..

[27] Fridovich I., Gilbert D.L. (1981). Superoxide Radical and Superoxide Dismutases. Oxygen and Living Processes.

[28] Yelumalai S., Giribabu N., Karim K., Omar S.Z., Salleh N.B. (2019). In vivo administration of quercetin ameliorates sperm oxidative stress, inflammation, preserves sperm morphology and functions in streptozotocin-nicotinamide induced adult male diabetic rats. Arch. Med. Sci..

[29] Flohé L., Günzler W.A., Packer L. (1984). Assays of glutathione peroxidase. Methods in Enzymology.

[30] Ahmed A.Y., Aowda S.A., Hadwan M.H. (2021). A validated method to assess glutathione peroxidase enzyme activity. Chem. Pap..

[31] Girotti M.J., Khan N., McLellan B.A. (1991). Early measurement of systemic lipid peroxidation products in the plasma of major blunt trauma patients. J. Trauma Acute Care Surg..

[32] Fusco R., Salinaro A.T., Siracusa R., D’Amico R., Impellizzeri D., Scuto M., Ontario M.L., Crea R., Cordaro M., Cuzzocrea S. (2021). Hidrox^®^ counteracts cyclophosphamide-induced male infertility through NRF2 pathways in a mouse model. Antioxidants.

[33] Zeng C., He L., Peng W., Ding L., Tang K., Fang D., Zhang Y. (2014). Selection of optimal reference genes for quantitative RT-PCR studies of boar spermatozoa cryopreservation. Cryobiology.

[34] Zeng C., Peng W., Ding L., He L., Zhang Y., Fang D., Tang K. (2014). A preliminary study on epigenetic changes during boar spermatozoa cryopreservation. Cryobiology.

[35] Mao S., Goodrich R.J., Hauser R., Schrader S.M., Chen Z., Krawetz S.A. (2013). Evaluation of the effectiveness of semen storage and sperm purification methods for spermatozoa transcript profiling. Syst. Biol. Reprod. Med..

[36] Goodrich R., Johnson G., Krawetz S.A. (2007). The preparation of human spermatozoal RNA for clinical analysis. Arch. Andr..

[37] Miyoshi N., Barton S.C., Kaneda M., Hajkova P., Surani M.A. (2006). The continuing quest to comprehend genomic imprinting. Cytogenet. Genome Res..

[38] Oliva R. (2006). Protamines and male infertility. Hum. Reprod. Upd..

[39] Wu Y., Zhao J., Xu C., Ma N., He T., Zhao J., Ma X., Thacker P.A. (2020). Progress towards pig nutrition in the last 27 years. J. Sci. Food Agric..

[40] Dong H.J., Wu D., Xu S.Y., Li Q., Fang Z.F., Che L.Q., Wu C.M., Xu X.Y., Lin Y. (2016). Effect of dietary supplementation with amino acids on boar sperm quality and fertility. Anim. Reprod. Sci..

[41] Lin Y., Li J., Wang K., Fang Z., Che L., Xu S., Feng B., Zhuo Y., Li J., Wu D. (2022). Effects of dietary L-leucine supplementation on testicular development and semen quality in boars. Front. Vet. Sci..

[42] Parsley M.A., Wilson M.E., Gall T.J., Ballard M.R.M. (2021). Effect of Stabilized Fish Oil Source on Sperm Quality and Production of Boars. Open J. Anim. Sci..

[43] Li J., Barranco I., Tvarijonaviciute A., Molina M.F., Martinez E.A., Rodriguez-Martinez H., Parrilla I., Roca J. (2018). Seminal plasma antioxidants are directly involved in boar sperm cryotolerance. Theriogenology.

[44] Vickram S., Rohini K., Srinivasan S., Veenakumari D.N., Archana K., Anbarasu K., Jeyanthi P., Thanigaivel S., Gulothungan G., Rajendiran N. (2021). Role of zinc (Zn) in human reproduction: A journey from initial spermatogenesis to childbirth. Int. J. Mol. Sci..

[45] Andriola Y.T., Moreira F., Anastácio E., Camelo F.A., Silva A.C., Varela A.S., Gheller S.M.M., Goularte K.L., Corcini C.D., Lucia T. (2018). Boar sperm quality after supplementation of diets with omega-3 polyunsaturated fatty acids extracted from microalgae. Andrologia.

[46] Zamora-Zamora V., Figueroa-Velasco J.L., Cordero-Mora J.L., Nieto-Aquino R., García-Contreras A.D.C., Sánchez-Torres M.T., Carrillo-Domínguez S., Martínez-Aispuro J.A. (2017). Conjugated linoleic acid supplementation does not improve boar semen quality and does not change its fatty acid profile. Vet. Mex..

[47] Barranco I., Padilla L., Tvarijonaviciute A., Parrilla I., Martinez E.A., Rodriguez-Martinez H., Yeste M., Roca J. (2019). Levels of activity of superoxide dismutase in seminal plasma do not predict fertility of pig AI-semen doses. Theriogenology.

[48] Ribas-Maynou J., Yeste M. (2020). Oxidative stress in male infertility: Causes, effects in assisted reproductive techniques, and protective support of antioxidants. Biology.

[49] O’Flaherty C., Scarlata E. (2022). Oxidative stress and reproductive function: The protection of mammalian spermatozoa against oxidative stress. Reproduction.

[50] Lazzarino G., Listorti I., Bilotta G., Capozzolo T., Amorini A.M., Longo S., Caruso G., Lazzarino G., Tavazzi B., Bilotta P. (2019). Water-and fat-soluble antioxidants in human seminal plasma and serum of fertile males. Antioxidants.

[51] Walke G., Gaurkar S.S., Prasad R., Lohakare T., Wanjari M. (2023). The impact of oxidative stress on male reproductive function: Exploring the role of antioxidant supplementation. Cureus.

[52] Donkin I., Barrès R. (2018). Sperm epigenetics and influence of environmental factors. Mol. Metab..

[53] Okada Y., Scott G., Ray M.K., Mishina Y., Zhang Y. (2007). Histone demethylase *JHDM2A* is critical for Tnp1 and *Prm1* transcription and spermatogenesis. Nature.

[54] McSwiggin H.M., O’doherty A.M. (2018). Epigenetic reprogramming during spermatogenesis and male factor infertility. Reproduction.

