# Supplementary material for: Impact of Supplemented Nutrition on Semen Quality, Epigenetic-Related Gene Expression, and Oxidative Status in Boars
Source: Animals (Basel). 2024 Nov 15;14(22):3297. doi: 10.3390/ani14223297 (PMC11591274; doi:10.3390/ani14223297)
Supplement: Supplementary file 1 [file animals-14-03297-s001.zip › animals-3280495-supplementary.pdf]

Table S1. Mean±SE and ANOVA *p*-values for the effect of supplementation on boar sperm quality parameters.

**Parameter** [Unit]	**Group**[1]	**Sampling moment**[2]				***p* Value**[3]		
		P1	P2	P3	P4	T	P	T x P
**Conc.** **[**10^6/ml**]**	CON	42.7±2.8^{Aa}	37.0±3.6^{Aa}	43.6±3.0^{Aa}	42.2±3.8^{Aa}	NS	NS	NS
	ESP	39.0±2.5^{Aa}	43.2±3.9^{Aab}	48.1±3.0^{Ab}	52.5±4.0^{Bb}			
**TM** [%]	CON	75.1±2.0^{Aa}	81.7±2.2^{Ab}	78.0±2.9^{Aab}	80.0±2.2^{Aab}	**	***	NS
	ESP	76.6±3.0^{Aa}	83.2±1.2^{Ab}	86.3±1.4^{Bc}	88.4±1.0^{Bc}			
**PM** [%]	CON	65.3±2.5^{Aa}	75.3±2.7^{Ab}	71.5±3.1^{Aab}	73.4±2.4^{Ab}	*	***	NS
	ESP	67.4±0.4^{Aa}	75.1±1.5^{Ab}	78.7±2.3^{Abc}	82.8±1.4^{Bc}			
**VCL** **(**μm/s**)**	CON	95.2±6.8^{Aa}	100.6±5.0^{Aa}	103.2±5.3^{Aa}	109.4±5.0^{Aa}	NS	***	NS
	ESP	99.3±6.2^{Aa}	101.6±6.0^{Aa}	115.1±5.5^{Aa}	130.5±6.8^{Bb}			
**VSL** **[**μm/s]	CON	34.2±1.7^{Aa}	44.0±1.6^{Ab}	40.7±1.5^{Ab}	42.7±1.1^{Ab}	*	***	NS
	ESP	40.1±2.8^{Aa}	43.9±1.3^{Aab}	43.1±.01^{Aab}	45.5±1.2^{Ab}			
**VAP** **[**μm/s**]**	CON	49.0±2.3^{Aa}	51.6±2.1^{Aab}	50.5±2.5^{Aab}	55.6±2.1^{Ab}	*	***	NS
	ESP	43.1±2.6^{Aa}	51.4±2.2^{Ab}	53.2±2.2^{Ab}	59.5±1.9^{Ac}			
**DCL** **[**μm**]**	CON	40.0±2.9^{Aa}	43.1±2.1^{Aa}	45.5±3.2^{Aab}	49.5±3.1^{Ab}	NS	***	NS
	ESP	41.1±2.6^{Aab}	41.2±1.7^{Aa}	47.6±2.3^{Abc}	54.2±3.0^{Ac}			

**DSL** [μm]	CON	14.3±1.3^{Aa}	16.1±0.4^{Aab}	15.9±0.2^{Aab}	16.7±0.7^{Ab}	NS	**	NS
	ESP	12.3±0.7^{Aa}	17.4±0.7^{Ab}	14.8±0.7^{Ac}	15.7±0.6^{Ac}			
**DAP** [μm]	CON	16.5±1.0^{Aa}	21.0±0.9^{Ab}	19.5±1.0^{Aab}	21.3±0.9^{Ab}	NS	***	NS
	ESP	18.5±1.4^{Aa}	19.5±0.5^{Aa}	20.5±1.0^{Aa}	23.4±0.9^{Ab}			
**ALH** [μm]	CON	0.93±0.07^{Aa}	0.88±0.06^{Aa}	1.00±0.08^{Aab}	1.08±0.08^{Ab}	NS	***	*
	ESP	0.91±0.06^{Aa}	0.89±0.04^{Aa}	1.06±0.05^{Ab}	1.18±0.08^{Abc}			
**BCF** [Hz]	CON	17.20±0.96^{Aa}	22.73±0.79^{Ab}	20.95±0.91^{Ab}	21.07±0.88^{Ab}	NS	***	NS
	ESP	18.52±1.68^{Aa}	21.02±0.59^{Aab}	22.28±1.24^{Abc}	23.87±0.80^{Bc}			
**HAC** [rad]	CON	0.28±0.02^{Aa}	0.33±0.02^{Aab}	0.33±0.02^{Aab}	0.36±0.02^{Ab}	NS	***	NS
	ESP	0.28±0.02^{Aa}	0.31±0.01^{Aa}	0.33±0.02^{Aa}	0.39±0.02^{Ab}			

[1]CON – control group; ESP – group supplemented with *Espermaplus*; [2]P1 – the day when *Espermaplus* supplementation started; P2 – the day after three-week period of *Espermaplus* supplementation; P3 – the day after the eight-week period of *Espermaplus* supplementation; P4 – the day after the twelve-week period of *Espermaplus* supplementation; [3]Significance was declared at p<0.05; T – treatment; P – period; T x P – interaction between treatment and period; Conc. – concentration of spermatozoa; TM – total sperm motility; PM – progressive sperm motility; VCL – curvilinear velocity; VSL – straight-line velocity; VAP – average path velocity; DCL – curvilinear distance; DSL – straight line distance; DAP – distance of average path; ALH – amplitude of lateral head displacement; BCF – beat-cross frequency; HAC – head activity; [abc]Different lowercase letters indicate statistically significant differences (p<0.05) within the same group at different moments; [ABC]Different uppercase letters indicate statistically significant differences (p<0.05) between groups at the same sampling moment. * - p<0.05; ** - p<0.01; *** - p<0.001; NS - no significance.
